# Virion stiffness regulates immature HIV-1 entry

**DOI:** 10.1186/1742-4690-10-4

**Published:** 2013-01-10

**Authors:** Hong-Bo Pang, Liron Hevroni, Nitzan Kol, Debra M Eckert, Marianna Tsvitov, Michael S Kay, Itay Rousso

**Affiliations:** 1 Department of Biochemistry, University of Utah School of Medicine, Salt Lake City, UT 84112-5650, USA; 2 Department of Structural Biology, Weizmann Institute of Science, Rehovot 76100, Israel; 3 Present address: Sanford-Burnham Medical Research Institute, La Jolla, CA 92037, USA; 4 Present address: Department of Physiology and Cell Biology, Faculty of Health Sciences, Ben-Gurion University, Beer-Sheva 84105, Israel

**Keywords:** HIV, Viral entry, Atomic force microscopy, Maturation, Stiffness

## Abstract

**Background:**

Human immunodeficiency virus type 1 (HIV-1) undergoes a protease-mediated maturation process that is required for its infectivity. Little is known about how the physical properties of viral particles change during maturation and how these changes affect the viral lifecycle. Using Atomic Force Microscopy (AFM), we previously discovered that HIV undergoes a “stiffness switch”, a dramatic reduction in particle stiffness during maturation that is mediated by the viral Envelope (Env) protein.

**Results:**

In this study, we show that transmembrane-anchored Env cytoplasmic tail (CT) domain is sufficient to regulate the particle stiffness of immature HIV-1. Using this construct expressed in *trans* with viral Env lacking the CT domain, we show that increasing particle stiffness reduces viral entry activity in immature virions. A similar effect was also observed for immature HIV-1 pseudovirions containing Env from vesicular stomatitis virus.

**Conclusions:**

This linkage between particle stiffness and viral entry activity illustrates a novel level of regulation for viral replication, providing the first evidence for a biological role of virion physical properties and suggesting a new inhibitory strategy.

## Background

The main structural component of an HIV-1 particle is the viral Gag polyprotein, which polymerizes to form a protein shell surrounded by a lipid membrane. Expression of Gag alone is necessary and sufficient for viral particle assembly and budding [1]. The viral surface contains the envelope protein (Env), which is synthesized as a precursor (gp160) that is cleaved by a cellular protease into receptor-binding (gp120) and transmembrane (gp41) subunits. gp120 and gp41 form a noncovalent complex that mediates viral entry [2,3]. The Env transmembrane subunit of HIV-1 and other lentiviruses has an unusually long (~150 amino acids) cytoplasmic tail (CT) domain compared to other retroviruses (~20-30 amino acids). Gag interacts with Env via CT, which aids Env localization to viral budding sites and efficient incorporation into virions [4-6].

During the viral lifecycle, a virion needs to meet several distinct demands—efficient membrane fusion during entry, particle disassembly to release genetic material, assembly during budding, and stability in the extracellular environment before entry into the next cell. HIV-1 virions initially emerge from infected cells as immature particles. These particles then undergo a maturation process induced by HIV-1 protease cleavage of Gag into several products including three structural proteins: MA, CA and NC [7]. Electron Microscopy (EM) shows that HIV-1 particles undergo a dramatic morphological change from a roughly spherically symmetric immature particle with a thick protein shell to a mature particle with a prominent conical core (capsid) formed by CA [8]. In mature virions, only MA remains associated with the viral membrane, creating a thin protein shell. Because of this striking morphological change and the requirement to address diverse needs throughout their lifecycle, we hypothesized that the physical properties of viral particles would also change during maturation. Atomic force microscopy (AFM) has proven to be uniquely informative for measuring the mechanical properties of viral particles under native conditions. We and others have used AFM to measure the physical properties of several viruses [9-12].

Using AFM, we determined that HIV-1 immature particles are ~14-fold stiffer than mature ones, reflecting a dramatic biophysical change during maturation that we call the “stiffness switch” [13]. Further studies determined that Env CT is required for the stiffness switch, as its deletion softens immature HIV-1 particles almost to the mature level [13]. Immature HIV-1 virions are not infectious due to both entry and post-entry (e.g., integration) defects [14,15]. Deletion of CT restores the entry activity of immature HIV-1 to the mature level [13-15]. These results suggest a strong inverse correlation between viral particle stiffness and entry activity. However, deletion of CT has been reported to induce conformational changes in gp120, which could affect viral entry activity [16]. Therefore, a cause and effect relationship between particle stiffness and viral entry has not yet been demonstrated, and the link between virion physical properties and biological function remains an open question.

**Figure 1:**
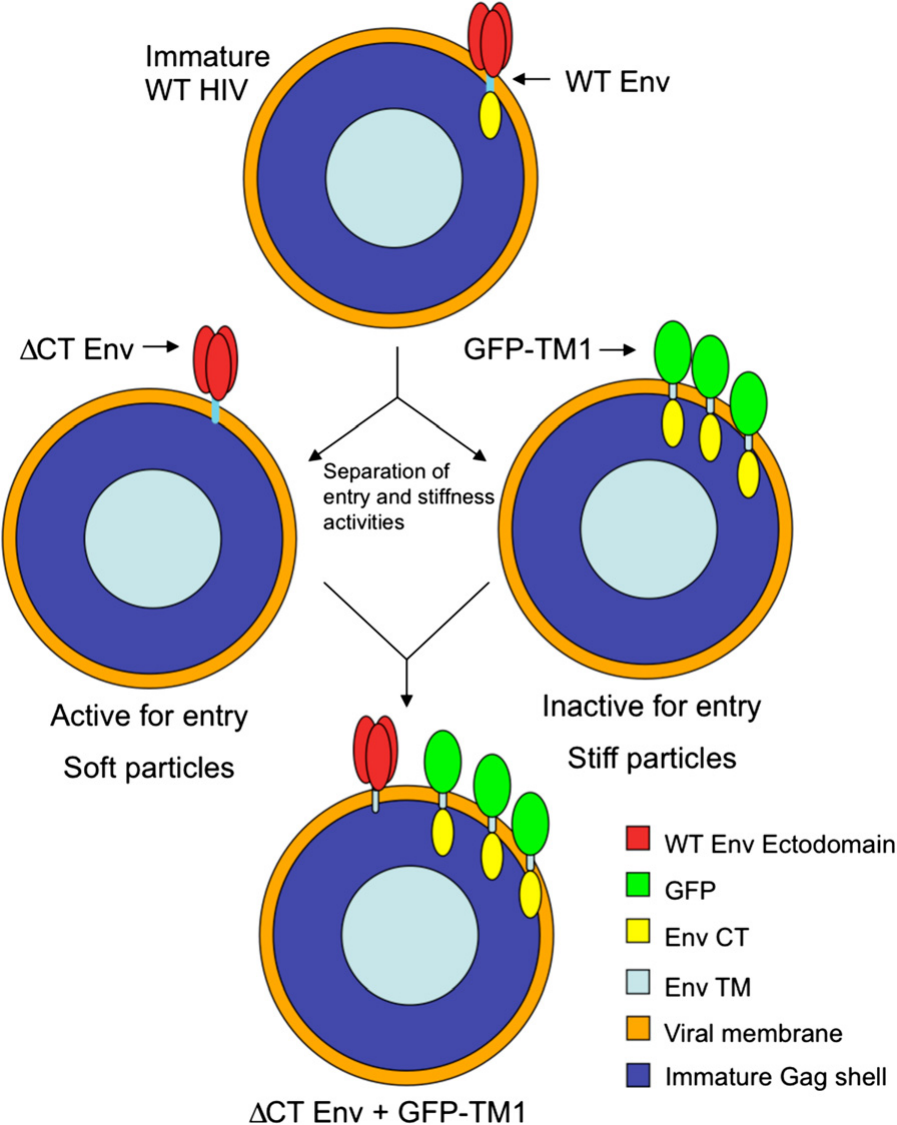
**Schematic of experimental strategy for separation of Env entry and stiffness activites.** A WT immature particle (top) contains the full Env protein, including ectodomain and CT. Immature virions bearing ΔCT Env (middle left) retain viral entry activity, but lose particle stiffness, while immature particles bearing GFP-TM1 (middle right) lack any entry activity due to their missing Env ectodomain, but retain particle stiffness. Titration of GFP-TM1 into immature particles containing ΔCT Env (bottom) allows measurement of how increasing particle stiffness affects entry activity.

In this study, we ask whether virion stiffness directly regulates its entry activity. To isolate the effect of particle stiffness on viral entry, we designed constructs that separate Env’s fusion and stiffness-mediating activities (Figure 1). Using these constructs, we show that membrane-anchored CT alone can stiffen immature HIV-1 particles in a concentration-dependent manner. Increasing particle stiffness inversely regulates viral entry mediated by either coexpressed HIV-1 ΔCT Env or an unrelated viral envelope protein (VSVg). This study provides the first direct evidence showing that particle stiffness regulates viral entry activity, linking a virion’s physical and biological properties. Our studies define a new regulatory level for viral replication and suggest a new strategy for the development of novel HIV-1 inhibitors.

## Results

**Figure 2:**
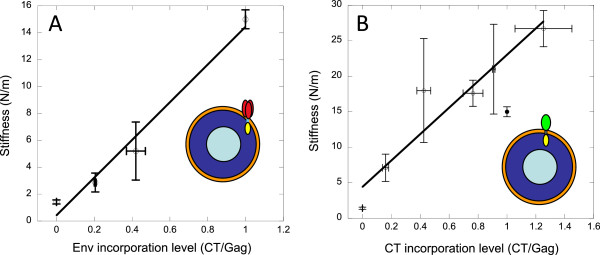
**Env and GFP-TM1 incorporation levels correlate with immature particle stiffness.** Different amounts of WT Env (**A**) or GFP-TM1 (**B**) were incorporated into immature HIV-1, and the particle stiffness of these viruses was measured using AFM. At least 16 particles were measured for each stiffness value, except the two highest GFP-TM1 points, which produced low particle yields (4 and 6 particles measured, respectively). Viral particle stiffness is plotted against Env or GFP-TM1 incorporation levels normalized to virus with highest level of incorporation (open circles, linear fit shown). The GFP-TM1 titration series was analyzed together with immature WT (filled circle). Error bars show SEM.

In order to measure the effect of Env incorporation levels on immature virion stiffness, we generated immature HIV-1 with increasing amounts of WT Env by titrating Env plasmid input during transfection. To produce immature virions, we use a viral genome containing Gag with mutated protease sites (immature genome), thus preventing the proteolytic processing of full-length Gag and viral maturation. WT Env was detected by anti-CT antibody and Env incorporation levels were calculated as a CT:Gag ratio. Mature particles are produced using a WT genome that contains WT Gag, which is cleaved by HIV protease during maturation. WT Env incorporation in immature HIV-1 gradually increases viral particle stiffness (Figure 2A).

### CT alone is sufficient to regulate immature viral particle stiffness

As we previously reported [13], HIV-1 Env, specifically the CT domain, is necessary to produce stiff immature particles. To discern whether CT alone can stiffen viral particles, we first deleted the Env ectodomain (all of gp120 and most of gp41), leaving the gp41 transmembrane (TM) and CT domains. This construct was poorly incorporated into virions, and introduction of a soluble trimeric coiled-coil ectodomain (to mimic trimeric Env) did not improve incorporation (data not shown). Next, we reasoned that replacing gp120 with a soluble globular protein like GFP might improve incorporation. Using GFP to replace the ectodomain, but leaving TM and CT intact (GFP-TM1) resulted in efficient incorporation (data not shown).

To investigate whether GFP-TM1 can stiffen viral particles similarly to WT Env, we generated immature particles with escalating amounts of GFP-TM1. As with Env, GFP-TM1 incorporation levels were calculated as a CT:Gag ratio. Increasing GFP-TM1 incorporation also gradually stiffens immature particles to beyond the WT immature level (Figure 2B). These data show that CT alone is sufficient to stiffen immature viral particles in a similar manner to WT Env.

### Particle stiffness regulates immature HIV-1 entry

Both viral stiffness and entry are controlled by Env. To investigate whether particle stiffness directly regulates viral entry, we separated Env’s stiffness-mediating and entry-inducing domains. GFP-TM1 stiffens viral particles without introducing entry activity (due to its missing ectodomain). ΔCT Env efficiently mediates entry, but contributes little to particle stiffness [13]. These constructs allow us to modulate virion stiffness as an independent variable to determine its effect on viral entry activity.

We produced immature virions with varying amounts of GFP-TM1 (to modulate stiffness) and a fixed amount of ΔCT Env (to provide entry activity). GFP-TM1 and ΔCT Env incorporation levels were measured by WB as CT:Gag and gp120:Gag ratios, respectively. The specific entry activities of viruses with different GFP-TM1 incorporation levels were measured by a viral fusion (BlaM) assay and normalized to corresponding ΔCT viruses with no GFP-TM1.

**Figure 3:**
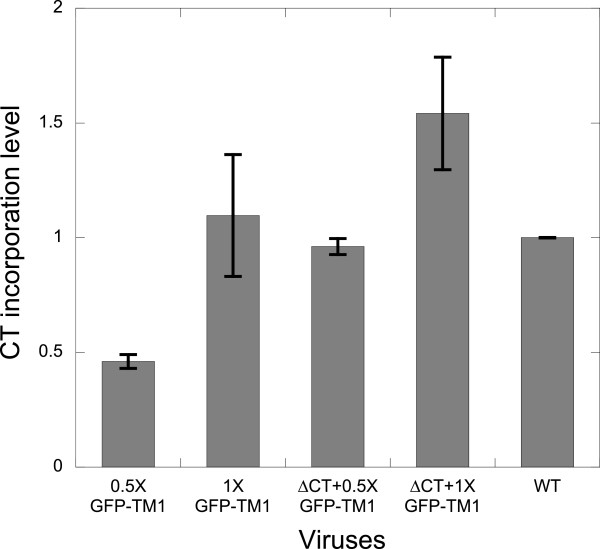
**ΔCT coexpression slightly increases GFP-TM1 incorporation.** GFP-TM1 was incorporated in immature HIV-1 with or without ΔCT Env. 1X corresponds to 100% GFP-TM1 plasmid input amount. For 0.5X or 1X GFP-TM1, ΔCT plasmid input was replaced by the same amount of an inert bacterial vector (pCDF-BS) to balance plasmid mass during transfection. Input amount of all other plasmids (i.e., genome and GFP-TM1) remain the same with the corresponding viruses bearing ΔCT. CT incorporation levels are normalized to that of immature WT. Error bars indicate the SEM.

**Figure 4:**
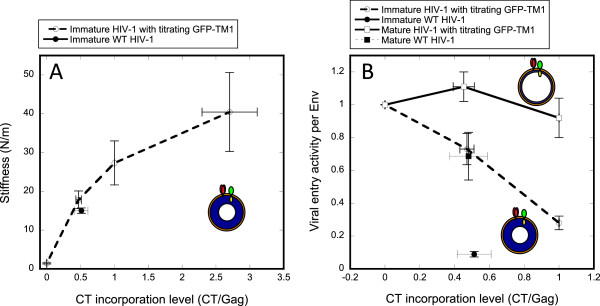
**Particle stiffness regulates immature HIV-1 entry.** Varying amounts of GFP-TM1 were incorporated into immature or mature HIV-1 bearing ΔCT Env (HXB2). The gp120 level represents all Env capable of mediating viral entry (ΔCT or WT Env). Error bars show SEM. Immature (orange dash) viruses bearing different amounts of GFP-TM1 were generated and analyzed together with immature WT (blue diamond) or mature WT (blue circle). (**A**) Virion stiffness (at least 19 particles were measured for each stiffness value) or (**B**) viral entry activity per Env normalized to the corresponding mature or immature ΔCT viruses.

Coexpression with ΔCT mildly increases GFP-TM1 incorporation relative to GFP-TM1 alone (Figure 3). As with GFP-TM1 alone, GFP-TM1 incorporation in the presence of ΔCT Env gradually increases immature particle stiffness (Figure 4A). As previously reported, Env with intact CT has little effect on mature particle stiffness [13]. Therefore, a GFP-TM1 titration series in mature virions serves as a control to isolate the effects of GFP-TM1 incorporation on viral entry independent of changing particle stiffness.

**Figure 5:**
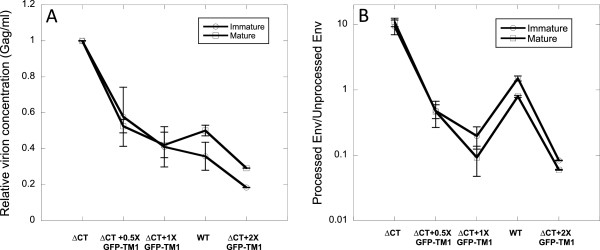
**GFP-TM1 incorporation reduces viral yield and gp160 processing.** Immature (circles) and mature (squares) HIV-1 bearing different levels of GFP-TM1 and ΔCT were generated together with WT virus. 1X stands for 100% GFP-TM1 plasmid input. Error bars indicate the SEM. **A**). Viral yield (Gag per ml) is normalized to the yield of ΔCT virus. **B**). Ratio of processed Env (gp120) to unprocessed Env (gp160 for WT Env; gp140 for ΔCT Env) is plotted.

As shown in Figure 4B, increasing GFP-TM1 incorporation greatly impairs viral entry activity in immature ΔCT particles, while causing only modest reduction in the corresponding mature particles. This result strongly suggests that GFP-TM1 incorporation affects viral entry activity through changing particle stiffness. Besides viral entry activity, we also observed that increasing GFP-TM1 incorporation reduces viral yield and gp160 processing (cleavage to gp120 and gp41) (Figure 5). Since gp160 processing is required for HIV-1 entry [7], this change could provide another explanation for GFP-TM1’s effect on viral entry. However, gp160 processing declines similarly in both the immature and mature states, suggesting that the immature-specific changes in entry activity are not due to changes in gp160 processing (Figure 5). Nevertheless, poor gp160 processing reduces gp120 incorporation, in some cases to a level below the detection limit. As a result, the entry activity for virus with 200% GFP-TM1 plasmid input could not be accurately quantified.

**Figure 6:**
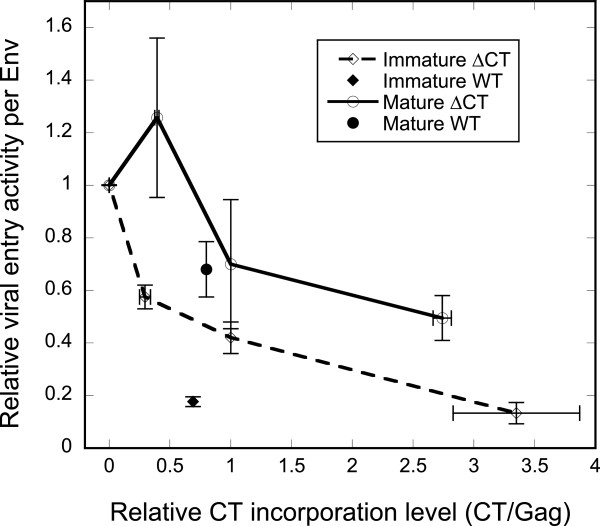
**GFP-TM1 incorporation reduces viral entry mediated by JRFL ΔCT Env.** Titrating amounts of GFP-TM1 were incorporated in immature (hollow diamond) or mature (hollow circle) HIV-1 bearing JRFL ΔCT Env. Immature (solid diamond) or mature (solid circle) JRFL WT viruses were also generated within the same batch as controls. CT incorporation levels (representing GFP-TM1 or WT Env) are normalized to those of the corresponding immature or mature viruses with 100% GFP-TM1 plasmid input. Specific viral entry activity is normalized to those of the corresponding immature or mature ΔCT viruses with no GFP-TM1. Error bars indicate the SEM.

In all of the above studies, we used HXB2, a commonly studied CXCR4-tropic laboratory HIV-1 strain [9,13]. We investigated whether the entry-suppressing effect of GFP-TM1 is general for HIV by studying JRFL, a primary CCR5-tropic strain, by coexpressing GFP-TM1 with JRFL ΔCT Env on mature or immature virions. An immature-specific loss of viral entry activity was observed with increasing GFP-TM1 incorporation, as seen with HXB2 ΔCT Env (Figure 6). In contrast to HXB2 ΔCT, increasing GFP-TM1 incorporation reduces JRFL ΔCT Env incorporation but not gp160 processing (data not shown). This observation further suggests that neither incorporation nor processing of Env contributes significantly to the immature-specific effect on viral entry induced by GFP-TM1 incorporation.

### Particle stiffness regulates viral entry of immature pseudovirions coated with VSVg

HIV-1 is generally thought to enter target cells at the plasma membrane, though a recent report suggests endocytosis as the primary entry route [17]. VSV, as a member of the *Rhabdoviridae* family, is unrelated to HIV-1 and enters target cells via endocytosis. VSV entry is mediated by its Env, VSVg [17-19]. Compared to HIV-1 Env, VSVg incorporates at much higher levels, requires no protease cleavage to mediate entry, and is unlikely to interact with GFP-TM1. Investigating the effect of GFP-TM1 incorporation on entry activity of HIV-1 pseudovirions coated with VSVg therefore provides an independent test of the relationship between particle stiffness and viral entry.

**Figure 7:**
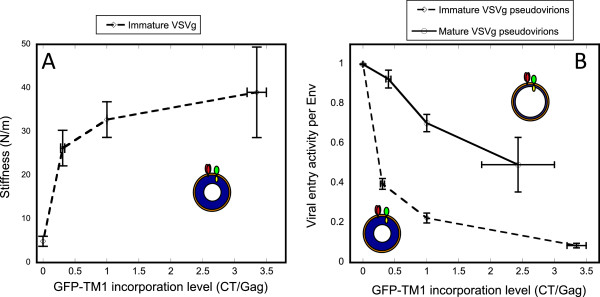
**Particle stiffness regulates immature entry mediated by VSVg.** Varying amounts of GFP-TM1 were incorporated into immature or mature HIV-1 bearing VSVg. Anti-VSVg antibody was used to quantify VSVg levels by WB. Viral entry activity was normalized by VSVg concentration for each virus to obtain its specific entry activity. Error bars indicate the SEM. **A**) Virion stiffness or **B**) specific viral entry activity is plotted against GFP-TM1 incorporation levels normalized to corresponding immature/mature virus with 100% GFP-TM1 plasmid input. Immature (orange open diamonds) and mature (blue open circles) HIV-1 bearing VSVg and GFP-TM1 are shown. Specific entry activity is normalized to the corresponding immature or mature virus with no GFP-TM1. At least 16 particles were measured for each stiffness value.

As with HIV-1 ΔCT Env, GFP-TM1 and VSVg were coexpressed and incorporated into mature or immature HIV-1 virions. WB was used to quantify GFP-TM1 and VSVg incorporation level as CT:Gag and VSVg:Gag ratio, respectively. Increasing GFP-TM1 incorporation increases particle stiffness in immature virions (Figure 7A). Similar to the effect on HIV-1 entry, increasing GFP-TM1 incorporation greatly reduces VSVg-mediated entry in immature virions with much less effect on mature virions (Figure 7B). VSVg incorporation level changes little with increasing GFP-TM1 incorporation (data not shown). These results further suggest that particle stiffness directly regulates viral entry.

### GFP-TM1 does not interact with viral Env

An important assumption of our study is that GFP-TM1 incorporation does not affect viral entry mediated by ΔCT Env or VSVg except by changing particle stiffness. As discussed earlier, GFP-TM1 cannot mediate viral entry itself, and GFP-TM1 is missing the critical self-associating residues of the gp41 ectodomain required for Env trimerization [20,21]. Nevertheless, there is still the possibility that GFP-TM1 interacts with ΔCT Env or VSVg.

**Figure 8:**
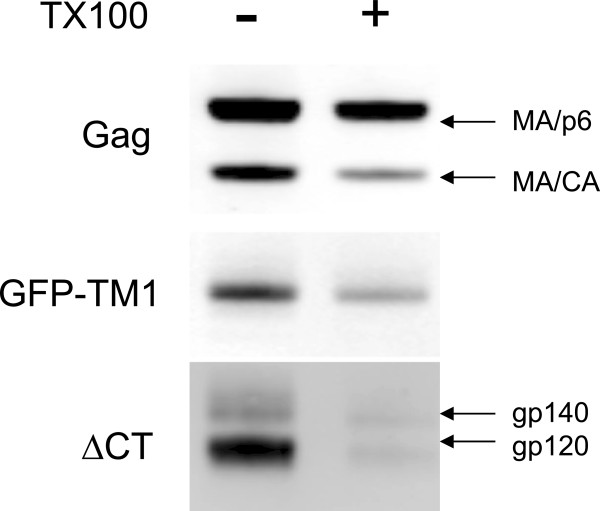
**GFP-TM1 does not interact with coexpressed viral Env.** Immature HIV-1 bearing both GFP-TM1 and JRFL ΔCT Env was treated with or without 0.5% TX100. Anti-CT, anti-gp120 and anti-CA antibodies were used to detect GFP-TM1, ΔCT Env (unprocessed gp140 or processed gp120), and Gag (MA/p6 - full length, MA/CA – full length missing p6), respectively.

To rule out this possibility, we used a non-ionic detergent, Triton X-100 (TX100), which does not dissociate WT Env from the immature Gag shell due to the noncovalent CT-Gag interaction, but removes Env with truncated CT [5,6]. Treating immature virions bearing both GFP-TM1 and JRFL ΔCT Env with TX100, GFP-TM1 remains associated with the Gag shell while almost all JRFL ΔCT Env dissociates (Figure 8). This result suggests that there is no specific interaction between GFP-TM1 and ΔCT Env. A similar result was observed for VSVg pseudovirions expressing GFP-TM1 (data not shown).

### Modest reduction of mature viral entry is likely due to over expression of exogenous protein on the viral membrane

**Figure 9:**
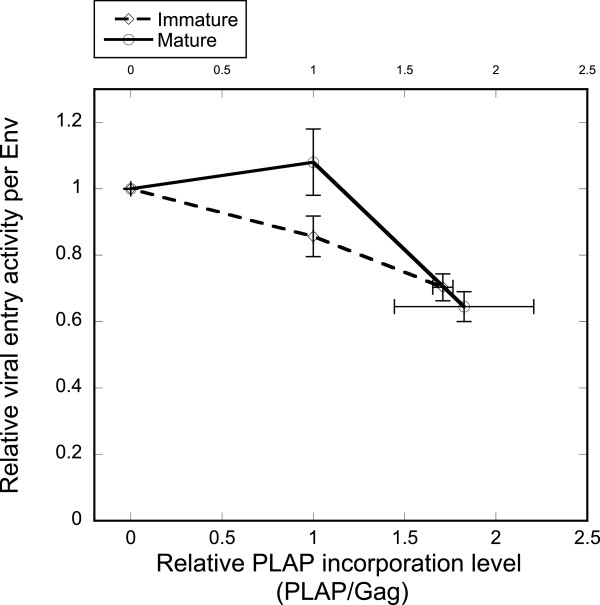
**PLAP incorporation causes mild reduction of viral entry activity in both mature and immature viruses.** Different amounts of PLAP were incorporated in the immature (diamond) or mature (circle) HIV-1 particles bearing ΔCT Env. PLAP incorporation level was calculated as a PLAP:Gag ratio normalized to virus with 100% PLAP plasmid input. Specific entry activity indicates viral entry activity per Env, normalized to that of virus with no PLAP input. Error bars indicate the SEM.

Although relatively immature-specific, GFP-TM1 does cause modest loss of viral entry activity in the mature state (Figures 4B and 7B). We hypothesize that this reduction is due to overexpression of an exogenous protein (e.g., GFP-TM1) on the viral membrane. To test this hypothesis, we employed another membrane protein, PLAP (human placental alkaline phosphatase), to see whether exogenous protein overexpression causes a similar modest reduction of viral entry. PLAP is a cell surface, glycosylphosphatidylinositol anchored protein, and is not normally present on the lymphoid cell surface [22]. As with GFP-TM1, we cotransfected ΔCT Env (HXB2 strain) with titrating PLAP plasmid levels to produce immature and mature HIV-1 virions. Increasing PLAP incorporation induces modest reduction of viral entry activity in both mature and immature virions, similar to the effect of GFP-TM1 incorporation on mature viral entry (Figure 9).

## Discussion

**Figure 10:**
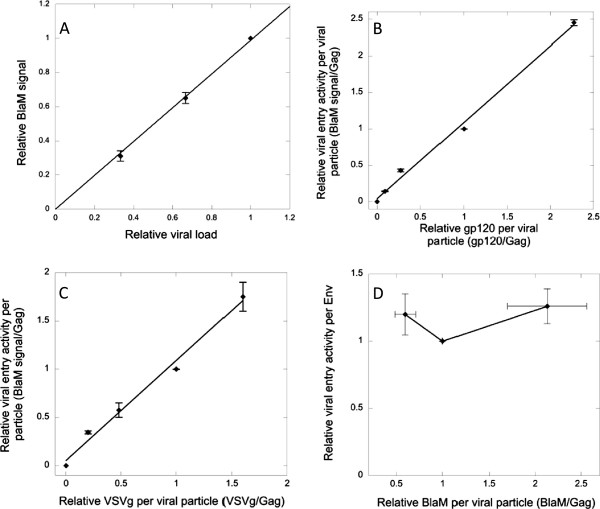
**Optimization of BlaM viral entry assay. A**). Immature ΔCT HIV-1 viruses. BlaM signal is plotted against relative amount of loaded virus. **B**). Immature HIV-1 bearing titrating amounts of ΔCT Env. Entry signal per virion is plotted against gp120 per virion (determined by WB), both normalized to virus with 100% ΔCT plasmid input. BlaM assay signal increases proportionally to gp120 concentration (linear fit). **C**). Immature HIV-1 bearing titrating amounts of VSVg. Entry signal per virion is plotted against VSVg per virion, both normalized to virus with 100% VSVg plasmid input. BlaM assay signal increases proportionally to VSVg concentration (linear fit). **D**). Immature HIV-1 bearing different levels of BlaM. Entry signal per virion is plotted against BlaM per virion, both normalized to virus with 100% BlaM plasmid input. 2–fold variation of BlaM concentration minimally changes the entry assay signal. Error bars indicate the standard error of the mean (SEM).

In this study, we investigated whether HIV-1 employs variation in particle stiffness as a novel regulatory mechanism for viral entry. Since both stiffness and viral entry are regulated by a single protein (HIV-1 Env), we separated the stiffness-regulating CT domain and entry-mediating Env ectodomain to independently modulate these two properties. A membrane-anchored CT construct, GFP-TM1, is sufficient to regulate immature particle stiffness in a similar manner to WT Env and showed no specific interaction with coexpressed entry-mediating Env (ΔCT Env or VSVg). Since GFP-TM1 incorporation showed an immature-specific effect on viral entry activity, we conclude that increasing particle stiffness is the most likely mechanism by which GFP-TM1 incorporation progressively inhibits immature viral entry. An important caveat for these results is that measured Env levels may also include microvesicle-associated Env, which may not be removed by our virion pelleting procedure. However, the linear relationship between viral entry activity and measured Env incorporation (Figure 10B) suggests a close correlation to virion-associated Env.

HIV-1 gp41 undergoes significant conformational changes to bring the viral and cellular membranes into close proximity to achieve membrane fusion. A likely mechanism by which particle stiffness affects viral entry activity is that stiffer particles may present a higher energy barrier to membrane deformation during fusion. Determining which step of entry is regulated by particle stiffness may help to further characterize this regulatory mechanism. We also showed that particle stiffness regulates viral entry mediated by the unrelated VSVg. Therefore, targeting particle stiffness (e.g., by preventing the stiffness switch during viral maturation), may represent a novel and general viral inhibitory strategy.

Gag is the major HIV structural protein, and the stiffness switch during HIV-1 maturation likely originates from changes in the Gag shell organization, which are visible by EM as a dramatic decrease in the thickness of the membrane-associated protein layer (thought to only consist of MA). MA relies on its N-terminal myristoyl group for membrane binding, and separation of Gag components from MA was shown to partially bury its myristoyl group, which destabilizes membrane binding and MA’s oligomeric structure [23,24].

Current estimates of the number of HIV-1 Env spikes present on the viral surface are very low (~10 trimers per virion), compared to up to 2,000 Gag molecules in the immature virion protein shell [25-28]. An interesting question is how so few CT domains can dramatically alter a global property like particle stiffness in ~100 nm particles. We propose two possibilities: 1) CT functions during assembly to seed a specific packing arrangement of the Gag lattice (analogous to seeding of different crystal forms), 2) CT functions via its interaction with Gag to stabilize the whole Gag shell. Future studies may distinguish between these two models by establishing the timing of CT’s regulation of particle stiffness (e.g., during budding vs. in released immature particles).

Interestingly, the fusion activity of mature HIV-1 is also blocked by a cholesterol-binding compound, AME [29]. Virions with CT truncations are AME-resistant [30]. HIV-1 virions are enriched in cholesterol as a consequence of their budding from lipid rafts [31,32], which raises the question whether lipid membrane components, especially cholesterol, are also important for particle stiffness. It is therefore tempting to speculate that AME may inhibit viral entry by stiffening mature particles, and AME resistance emerges by severing of the link between Env CT and Gag (via CT truncation).

Ultimately, mapping of the CT determinants involved in mediating stiffness and structural studies of the Gag/CT interaction will be required to reveal how CT affects the overall Gag shell organization. Future studies will also examine why HIV regulates entry activity via particle stiffness. Possibilities include prevention of viral entry until particle maturation is complete (to avoid non-productive entry, as suggested by [15]) or use of the immature virion as a more durable particle to facilitate distant spread of infection.

## Conclusions

Overall, these results demonstrate the first direct linkage between a viral physical property and its biological activity. Future mechanistic and functional studies of viral particle stiffness may enable the design of novel entry inhibitors that exploit this linkage.

## Methods

### Plasmids

Plasmids were obtained or constructed as follows (summarized in Table 1): ΔEnv HIV-1 genome vector containing an inactivating integrase mutant (DHIV3-GFP-D116G [33], provided by V. Planelles), HIV-1 Env expression vector (pEBB-HXB2 [34], provided by B. Chen), VSVg expression vector (phCMV-VSV-G [35,36], provided by W. Sundquist), Env expression plasmid for JRFL strain (pCAGGS-JRFL-Env WT and ΔCT, provided by J. Binley [37]) and vector expressing Vpr-ß-lactamase (BlaM-Vpr) fusion protein, pMM310 [14]. Human placental alkaline phosphatase (PLAP) expressing vector (pCMV-SPORT6) was from ATCC. Immature particles were generated by cloning Gag with all PR cleavage sites mutated (pNL-MA/p6 [14], provided by C. Aiken) into the ΔEnv Int − HIV-1 genome, while mature particles were produced using an HIV-1 genome vector with wild-type (WT) cleavage sites. ΔCT HXB2 Env (Δ147 [38]) was provided by E. Hunter and cloned into pEBB-HXB2.

**Table 1:** Plasmids used in this study

**ΔEnv HIV-1 genome vector**	**DHIV3-GFP-D116G**
Viral Env expression vectors	pEBB-HXB2
	pEBB-HXB2 ΔCT
	phCMV-VSVg
	pCAGGS-JRFL-Env
	pCAGGS-JRFL-Env ΔCT
β-lactamase expression vector	pMM310
GFP-TM1 expression vector	pEBB-HXB2 GFP-TM1
PLAP expression vector	pCMV-SPORT6 PLAP

To construct GFP-TM1, the GFP (green fluorescent protein) gene was obtained from pET9a-GFP-C37 [39] by PCR using a 5′ KpnI-containing primer (5′-tctgggtacctagctctggcatggtgagcaagggcgagg) and a 3′ SacI-containing primer (5′-ctcgaggagctcttgtacag). The gp41 TM + CT fragment was obtained from pEBB-HXB2 by PCR using 5′ SacI-containing primer (5′-caatgagctctggcggttggaattggtttaacataacaaattgg) and 3′ BamHI-containing primer (5’-gtcccagataagtgctaaggatc). These two PCR products were annealed at the SacI digestion site. The generated GFP-TM1 fusion fragment was ligated into pEBB-HXB2 to replace the corresponding KpnI-BamHI fragment, which includes Env residues from V44 to L681 (HXB2 numbering).

### Viral preparation and analysis

Pseudovirion particles were produced by cotransfection of 293T cells with ΔEnv Int- HIV-1 genome vector, an Env-expressing vector (WT or ΔCT), pMM310, and pEBB-GFP-TM1. To generate control immature or mature WT virus, 2.5 μg of total DNA (1.23 μg genome vector, 0.819 μg Env expressing vector, and 0.45 μg pMM310) was transfected into ~10^6^ cells using 10 μg polyethylenimine (PEI, Sigma). In most experiments, the amount of only one functional plasmid (Env-expressing vector, pMM310 or pEBB-GFP-TM1) is titrated, and an inactive balancer plasmid is added to keep total plasmid concentration constant in a titration series. The amount of each plasmid used in the control transfection (described above) is defined as 100%. 100% GFP-TM1, VSVg or PLAP input is the same as that of WT or ΔCT Env (0.819 μg). Unless otherwise indicated, the same amount of genome vector (1.23 μg) and pMM310 vector (0.45 μg) was used for all transfections. For Env/GFP-TM1 cotransfections, the Env input was kept constant at 100%, while GFP-TM1 levels were titrated. Media was changed 6 h after transfection to avoid PEI toxicity. Supernatants containing secreted viral particles were collected 30 h post-transfection and filtered through 0.2 μm Acrodisc syringe filters (Pall). Each series of viruses prepared on the same day is defined as one “batch”.

For western blot (WB) analysis of viral concentration and Env incorporation level, virus supernatants were purified by centrifugation through a sucrose cushion (20% sucrose in TNE buffer: 0.1 M NaCl, 1 mM EDTA, 10 mM Tris, pH 7.6) at 20,000 X g for 90 min at 4°C. A caveat of this method is that pelleted samples may also include vesicle-associated Env, which is difficult to distinguish from virion-associated Env. Since intact virions are needed to incorporate BlaM-Vpr, this caveat does not influence entry activity measurements, but may affect the accuracy of measured Env incorporation levels. The pellet was resuspended in SDS-PAGE reducing sample buffer and resolved by SDS-PAGE. WB was developed using rabbit polyclonal anti-CA and anti-VSVg (provided by W. Sundquist), mouse monoclonal anti-gp41 CT antibody (obtained from Chessie 8 hybridoma contributed by G. Lewis, NIH AIDS Research and Reference Reagent Program (ARRRP)), and sheep polyclonal anti-gp120 (contributed by M. Phelan, ARRRP). Secondary antibodies are goat anti-rabbit (IRDye 680, Li-Cor), donkey anti-mouse (IRDye 800, Li-Cor) and Rabbit anti-sheep (IRDye 800, Rockland). Blots were quantified using a Li-Cor Odyssey infrared scanner. GFP-TM1 and WT Env incorporation levels per virion were calculated as a CT:Gag ratio. ΔCT Env and VSVg incorporation levels were calculated as gp120:Gag and VSVg:Gag ratios, respectively. PLAP was detected by rabbit monoclonal anti-PLAP antibody (Abcam, ab16695).

### AFM measurement and analysis

For AFM measurements, virus-containing supernatant was first concentrated as previously described [40]. Briefly, the filtered viral supernatant was pelleted onto a 5 ml cushion of OPTI-PREP (Iodixanol, Sigma) in a SW-28 rotor (21,000 rpm, 90 min, 4°C). ~90% of the upper layer supernatant was then aspirated, and the lower layer supernatant was collected with a syringe, combined with 3–5 volumes of TNE buffer, and concentrated by centrifugal ultrafiltration with a MWCO of 100,000 (Vivaspin 20, Sartorius) three times at 3,000 X g.

For AFM imaging and force measurements, virus particles were attached to HMDS-coated microscope glass slides using previously described methods [13,40]. All AFM experiments were carried out using a Bioscope with a Nanoscope IV controller (Veeco) equipped with a dimension XY closed loop scanner mounted on an inverted optical microscope (Axiovert 200 M, Carl Zeiss AG). Images of virus particles were acquired in AFM tapping mode in a fluid environment (TNE buffer). Pyramidal silicone nitride probes either mlct (K = 1 N/m, Veeco) or NSC35 (K = 3-7 N/m, Micromasch) were used. The spring constants of the DNP probes were determined experimentally by measuring thermal fluctuations [41]. Since the amplitude of the NSC35 thermal fluctuations was too low, we used the method of Sader et al. [20] to determine their spring constants.

Virus stiffness was determined based on indentation type experiments, as previously described [9,13]. Briefly, for each indentation measurement, ~100 force-distance (FD) curves were performed at a scan rate of 0.5 Hz. Viral stiffness was derived mathematically from the slope of the FD curve. The stiffness of the virus was computed according to Hooke’s law on the assumption that our experimental system can be modeled as two springs (the virus and the cantilever) arranged in series. In this study, virus particles had a relatively wide range of point stiffnesses, which required using several types of cantilevers having different spring constants. In order to reduce error in the virus calculated point stiffness, we prefer that the measured point stiffness is not larger than 70% of the cantilever spring constant. If the majority of virus measured point stiffness values are larger than this threshold we use a stiffer cantilever to measure these particles. Particles are selected for measurement based on their appearance in AFM imaging in tapping mode (roughly round particles with 80–120 nm height and little background debris near them). Particles are excluded if particle stiffness rises or drops dramatically during measurement (reflecting poor virion attachment to the surface or irreversible “breaking”, respectively).

### *ß-lactamase* (BlaM) assay for viral entry measurement

The viral entry assay was performed as described [13]. Briefly, viruses mixed with DEAE-Dextran (4 μg/ml) were added onto HOS-CD4-CXCR4 cells (provided by B.Chen), followed by centrifugation at 1800 X g for 30 min at 4°C and then incubated at 37°C for 2 h. After aspiration of unbound viruses, 1 μM ß-lactamase substrate solution (CCF2-AM, Invitrogen) was incubated with cells at 13°C for 17 h. Uncleaved and cleaved CCF2-AM have emission peaks of 520 nm (green) and 447 nm (blue), respectively, under 409 nm excitation. Fluorescent signals from both channels were detected using an Olympus MVX10 fluorescent microscope and quantified using ImageJ software. The ratio of blue vs. green fluorescent signal was calculated as the BlaM assay signal. Each assay was performed in triplicate and their BlaM assay signals were averaged as the entry signal. In this assay, entry signal increases proportionally to the amount of virus added (Figure 10A). gp120 represents functional HIV-1 Env that is cleaved by cellular proteases and capable of mediating viral entry. To investigate the relationship between gp120 concentration and BlaM assay signal, immature viruses bearing different amounts of HIV-1 ΔCT Env were generated and measured for their entry activities. Entry signal per viral particle also increases proportionally with the amount of processed Env (gp120) per viral particle, showing that the BlaM assay signal depends on the processed Env concentration (Figure 10B).

Therefore, viral entry activity was normalized to gp120 levels for each GFP-TM1 titration series. The specific entry activity we report here represents the viral entry activity per processed Env. Relative viral entry activity was calculated by normalization of the entry activity of each virus to that of virus without GFP-TM1. For all measurements, the mean entry activities from at least two independent batches assayed separately are reported.

The BlaM assay signal also increases proportionally to VSVg incorporation level (Figure 10C), so VSV-specific entry activity was also normalized to VSVg levels. Similarly, a varying amount of pMM310 was used during transfection to create a series of viruses bearing titrating amounts of BlaM (detected by rabbit polyclonal anti-BlaM (Chemicon/Millpore)). 2-fold variation of BlaM incorporation level only slightly changed the BlaM assay signal (Figure 10D). Since anti-BlaM Western blots showed less than 2-fold variation in BlaM incorporation levels for all our GFP-TM1 titrating viruses (data not shown), BlaM incorporation level was not considered during normalization.

To investigate whether we can compare the entry activity of viruses from different batches, normalized entry activities of immature VSVg pseudovirions without GFP-TM1 were measured and found to be very similar between different batches (data not shown). Therefore, the entry activity of viruses is reported as a relative value to that of virus with no GFP-TM1 within each series.

### Triton X-100 (TX100) treatment

TX100 treatment of HIV-1 particles was performed as described previously [6]. Briefly, virus-containing supernatant was first concentrated using the sucrose cushion method described above. The pellet was then resuspended into 0.5% TX100 in TNE buffer and incubated at 4°C for 30 min before centrifugation in a Beckman TLA-55 rotor at 45,000 rpm for 30 min. After centrifugation, the pellet was resuspended in SDS-PAGE reducing sample buffer and analyzed by Western blot. WB was developed using primary antibodies (rabbit anti-CA, mouse anti-gp41 CT and sheep anti-gp120) and secondary antibodies (goat anti-rabbit, donkey anti-mouse and rabbit anti-sheep) described above and quantified using a Li-Cor Odyssey infrared scanner.

## Competing interests

The authors declare that they have no competing interests.

## Authors’ contributions

HBP, DME, MSK, and IR designed the experiments. HBP and DME performed biochemistry and virology experiments. LH, NK, and MT performed AFM measurements. HBP, LH, NK, DME, MSK, and IR performed data analysis. HBP, MSK, and IR wrote the manuscript. All authors reviewed and approved the manuscript.
